# Is the novel amyloid-β tetramer fold a stable conformation?

**DOI:** 10.1186/1758-2946-5-S1-P10

**Published:** 2013-03-22

**Authors:** Eileen Socher, Anselm HC Horn, Heinrich Sticht

**Affiliations:** 1 Bioinformatik, Friedrich-Alexander-Universität Erlangen-Nürnberg, Erlangen, 91054, Germany

## 

In the pathogenesis of Alzheimer's disease (AD), the most common neurodegenerative disorder, the amyloid-β (Aβ) peptide plays a key role. Originally, the Aβ fibrils were postulated to be the neurotoxic agents for a long time, because an increased presence of extracellular amyloid plaques, composed primarily of insoluble Aβ fibrils, is found in the brain of affected patients. Recent studies, however, showed a higher cytotoxicity for small Aβ oligomers than for the Aβ fibrils so that these soluble Aβ oligomers are moving to the centre of interest now [1,2].

Because of the unstable and noncrystalline nature of these species, obtaining structural information for small oligomers is an experimentally challenging task. Novel structural insight was obtained from a recent crystal structure of a tetramer formed by the amyloidogenic residues 18-41 of the Aβ peptide. To enhance stability, this fragment was genetically engineered into the CDR3 loop region of a shark Ig single variable domain antibody [3].

Since the respective crystal structure is stabilized by the antibody moiety, we investigated, whether the respective topology also represents a stable fold for the isolated Aβ-peptide.

We performed molecular dynamics simulations in explicit solvent for the isolated tetrameric amyloid-β fragment in two different lengths (17-40 and 17-42) and the derived dimer and monomer structures. In contrast to Aβ17-40, we observed a stable dynamical behaviour for the tetramer of Aβ17-42: the extension of the antiparallel β-sheet through the residues 41 and 42 is responsible for the enhanced structural stability.

In summary, our results suggest that the novel tetrameric structure represents a stable oligomer conformation for the longer and more neurotoxic Aβ42 species and thus could be a new target in rational drug design aiming at the prevention of toxic oligomer formation.
